# Enzyme-Free Glucose Biosensors Based on MoS_2_ Nanocomposites

**DOI:** 10.1186/s11671-020-3285-3

**Published:** 2020-03-12

**Authors:** Weijie He, Yixuan Huang, Jiang Wu

**Affiliations:** Institute of Fundamental and Frontier Sciences, University of Electronic Science and Technology of China, Chengdu, 610054 People’s Republic of China

**Keywords:** Glucose biosensors, Two-dimensional materials, Enzyme-free, Electrochemical

## Abstract

High-performance glucose biosensors are highly desired for healthcare. To meet these demands, glucose biosensors, particularly enzyme-free glucose biosensors, have received much attention. Two-dimensional materials, e.g., graphene, with high surface area, excellent electrical properties, and good biocompatibility, have been the main focus of biosensor research in the last decade. This review presents the recent progress made in enzyme-free glucose biosensors based on MoS_2_ nanocomposites. Two different techniques for glucose detections are introduced, with an emphasis on electrochemical glucose biosensors. Challenges and future perspectives of MoS_2_ nanocomposite glucose biosensors are also discussed.

## Introduction

Glucose concentration in the human blood is an important health indicator. For example, healthy people typically have a blood glucose level around 3.9–6.1 mM (1 mM = ∼ 18 mg/dL), and glucose concentrations outside of this range may indicate kidney dysfunction, diabetes, etc. [1]. Driven by the ever-increasing demand for healthcare, many efforts have been devoted to enzymatic glucose biosensors based on glucose oxidase (GO_*x*_) since the report of enzyme electrode by Updike and Hicks in 1967 [2]. Despite the simplicity, efficiency, high sensitivity, and selectivity of enzymatic glucose biosensors, two major challenges, i.e., high cost and instability, still cannot be satisfactorily addressed. This is because enzymatic glucose sensors use enzymes, such as GO_*x*_ to detect glucose indirectly, which often involves expensive catalysts and complicated device construction, and thus, relatively high cost [3, 4]. In addition, enzymes like GO_*x*_ are vulnerable to temperature, humidity, pH, and nonphysiological chemicals due to their intrinsic thermal and chemical instability [5, 6]. The immobilization of GO_*x*_ on an electrode surface often adds another layer of difficulty in fabricating enzymatic glucose sensors with good stability and reproducibility [7, 8].

Thanks to the development of the Internet of Things, sensors of low cost and high reliability have attracted increasingly more attention. In the pursuit of glucose sensors that meet these demands, enzyme-free electrochemical biosensors have gained popularity due to a number of advantages, including simplicity, high sensitivity, and stability [9–12]. Enzyme-free electrochemical biosensors directly detect glucose via electrocatalytic oxidation, which avoids the use of costly enzyme as well as improves stability in ambient conditions. Such biosensors are expected to open new opportunities for incorporation with portable devices and real-time glucose detection [13]. The key to realize practical enzyme-free glucose sensors is inexpensive, reliable, biocompatible, and abundant catalysts. To this goal, nanocomposites, e.g., composites of two-dimensional materials and nanoparticles, have been widely adopted as biosensor electrode materials [14]. In particular, bioelectronics based on two-dimensional (2D) materials becomes an exciting new interdisciplinary field, owing to many unique physical and chemical properties of 2D materials, including large specific surface area, excellent conductivity, and facile synthesis. For instance, the large specific surface area of 2D materials enables easy surface functionalization via hybridization. The high conductivity allows efficient charge transfer and collection in 2D materials. Among various 2D materials, graphene and its derivatives are undoubtedly the most studied material in biosensors [15]. Another type of 2D materials that has been widely explored in electronics and optoelectronics also show merits of being bioelectronics materials. Transition metal dichalcogenides (TMDs), especially molybdenum disulfide (MoS_2_), possess similar advantages of large specific surface area, chemical inertness, and surface functionality. Intercalation of foreign ions or molecules into TMD nanosheets can be readily achieved given their unique atomic structure [16]. An important property that makes MoS_2_ stand well out from other 2D materials is its high catalytic activity arises from exposed edges [13]. However, the disadvantage of MoS_2_ is also quite obvious. Compared with graphene, 2D MoS_2_ sheets have much lower electrical conductivity. Re-stacking of MoS_2_ nanosheets further limits the charge transfer as well as active reaction sites.

Marginal efforts have been made in enzyme-free glucose sensors based on 2D MoS_2_ until the very recent years. The poor performance of MoS_2_-based electrochemical devices has been well coped with by using a number of methods that successfully solved the low intrinsic conductivity. A few MoS_2_-based electrochemical glucose biosensors have been reported with performance exceeding its graphene counterparts [17]. Apart from electrochemical biosensors, low-cost non-electrochemical methods have also been recently studied for 2D MoS_2_, by taking advantage of the progress of MoS_2_ made in electronics and optoelectronics [18]. In this review, we summarize the recent advances in MoS_2_-based glucose biosensors. Particular attention is given to MoS_2_-based electrochemical glucose biosensors, which are described in the “Electrochemical Glucose Biosensor-Based MoS_2_ Nanocomposites” section. In the “High Sensitivity Glucose Detection Using MoS_2_ Field-Effect Transistors” section, MoS_2_ field-effect transistors for glucose detection of glucose are briefly introduced. Finally, the conclusion and future perspectives of MoS_2_ nanocomposite glucose biosensors are presented.

## Electrochemical Glucose Biosensor-Based MoS_2_ Nanocomposites

For a few decades, metals or alloys have been the main catalyst options for the direct electrocatalytic oxidation of glucose [19]. In the last decade, two-dimensional materials with large surface area as well as unique chemical and physical properties open new opportunities for many fields including electrochemical sensing, energy storage, and electronics [20]. In terms of electrochemical biosensing, nanocomposites of different 2D materials and catalysts show clear advantages over traditional catalysts. The synergistic coupling between these materials, namely synergistic effects, can lead to distinct enhancement in catalytic activity [21]. A good number of such nanocomposites, particularly those based on graphene or graphene derivatives, have been developed and applied to enzyme-free glucose sensors. Layered MoS_2_ is expected to possess similar advantages as it shares the material properties of graphene. Particularly, layer MoS_2_ nanosheets possess a large number of edges, which, similar to functionalized graphene sheets, act as active sites for catalytic reactions [22, 23].

Indeed, Huang et al. synthesized MoS_2_ nanoflowers by a hydrothermal method [24]. A glassy carbon electrode modified with the MoS_2_ nanoflowers and chitosan/Au nanoparticle composites showed distinct overpotential reduction for bisphenol A oxidation. The nanocomposite sensor showed an efficient electrocatalytic oxidation of bisphenol A as evidenced by the significantly increased current in the cyclic voltammograms. A good linear detection range from 0.05 to 100 μM is obtained for bisphenol A sensing. Also, a very detection limit of 5 nM is estimated. This work clearly demonstrated the excellent electrocatalytic activity and synergistic effects of Au/MoS_2_ nanocomposites. Similarly, MoS_2_-based nanocomposites have been used for enzyme-free glucose detection. MoS_2_ flowers with a large surface area were synthesized by a hydrothermal method using cetyltrimethylammonium bromide (CTAB) as a surfactant [25]. The morphology of the microflowers can be controlled by the pH of the reaction solution, concentration of CTAB surfactant, and annealing temperature. The MoS_2_ microflowers obtained at an annealing temperature of 500 °C showed good crystalline quality and hence improved charge transfer. Interestingly, electrochemical enzyme-free glucose sensing tests showed that the MoS_2_ microflower electrode without any functionalization can offer a high sensitivity of 570.71 μA mM^−1^ cm^−2^. Additionally, the sensor shows a wide linear detection range of up to 30 mM.

The synergistic effects of MoS_2_ catalysts doped or hybridized by foreign metals, such as Cu, Ni, Co, and Fe, also hold true for enhanced electrochemical catalysis of glucose. Huang et al. managed to combine the advantages of both the strong electrocatalytic activity of copper for glucose oxidation and the large surface area and active edge sites of MoS_2_ nanosheets [26]. The Cu nanoparticles decorated MoS_2_ nanosheets showed electrocatalytic activity towards glucose oxidation. A high sensitivity of 1055 μA mM^−2^ cm^−2^ and a linear detection range of up to 4 mM have been reported for the Cu/MoS_2_ nanocomposite glucose sensor. The sensitivity nearly doubled the value measured from the MoS_2_ microflower electrode. The sensor also demonstrated good selectivity in detecting glucose against uric acid, ascorbic acid, and dopamine. The interference current caused by these chemicals is only about 2.1–5.2% of that from glucose, and such a low interference current level can be considered negligible at physiological concentration.

Another attractive candidate is nickel (Ni), which has been extensively studied for Ni/graphene hybrids. Similar to Cu, Ni is also an earth-abundant metal. The redox couple of Ni^3+^/Ni^2+^ offers an impressive catalytic activity in alkaline media. Therefore, Huang et al. used MoS_2_ nanosheet as catalyst support to immobilize Ni nanoparticles [27]. MoS_2_ nanosheet was synthesized from MoS_2_ powder in ethanol/water mixed-solvent via liquid exfoliation. Ni nanoparticles were reduced on MoS_2_ nanosheet by heating a MoS_2_ nanosheet–ethylene glycol solution at 60 °C for 1 h after an addition of NiCl_2_·6H_2_O precursor and N_2_H_4_·H_2_O and NaOH solutions. A glucose sensor electrode was prepared by depositing Ni-MoS_2_ hybrid on a glassy carbon electrode. Cyclic voltammogram of the Ni/MoS_2_ hybrid-modified electrode clearly revealed glucose oxidization with a higher current than a Ni-modified reference electrode. The improved electrocatalytic activity was attributed to more active sites on MoS_2_ nanosheets as well as reduced Ni nanoparticle aggregation on a 2D material support. The amperometric results confirmed a good linear detection range up to 4 mM, a high sensitivity of 1824 μA mM^−1^ cm^−2^, and a low detection limit of 0.31 μM at a signal/noise ratio of 3 (S/N = 3). Compared with the Cu/MoS_2_ nanocomposite glucose sensor, there is further improvement in sensitivity by using Ni/MoS_2_ nanocomposites. The impact of the interfering species, including dopamine, ascorbic acid, and uric acid, on glucose sensing was also found to be marginal. More importantly, the sensor showed good reproducibility and high stability. A negligible 3.4% reduction in the response of the sensor was measured after ambient storage for 4 weeks. In addition, Anderson et al. reported a highly sensitive non-enzymatic glucose biosensor by incorporating colloidal silver nanoparticles with MoS_2_ [28]. The introduction of Ag nanoparticles was to address the intrinsic poor conductivity of MoS_2_. An excellent sensitivity of 9044.6 μA mM^−1^ cm^−2^ and a low detection limit of 0.03 μM were reported. However, the linear detection range is only up to 1 mM.

**Fig. 1:**
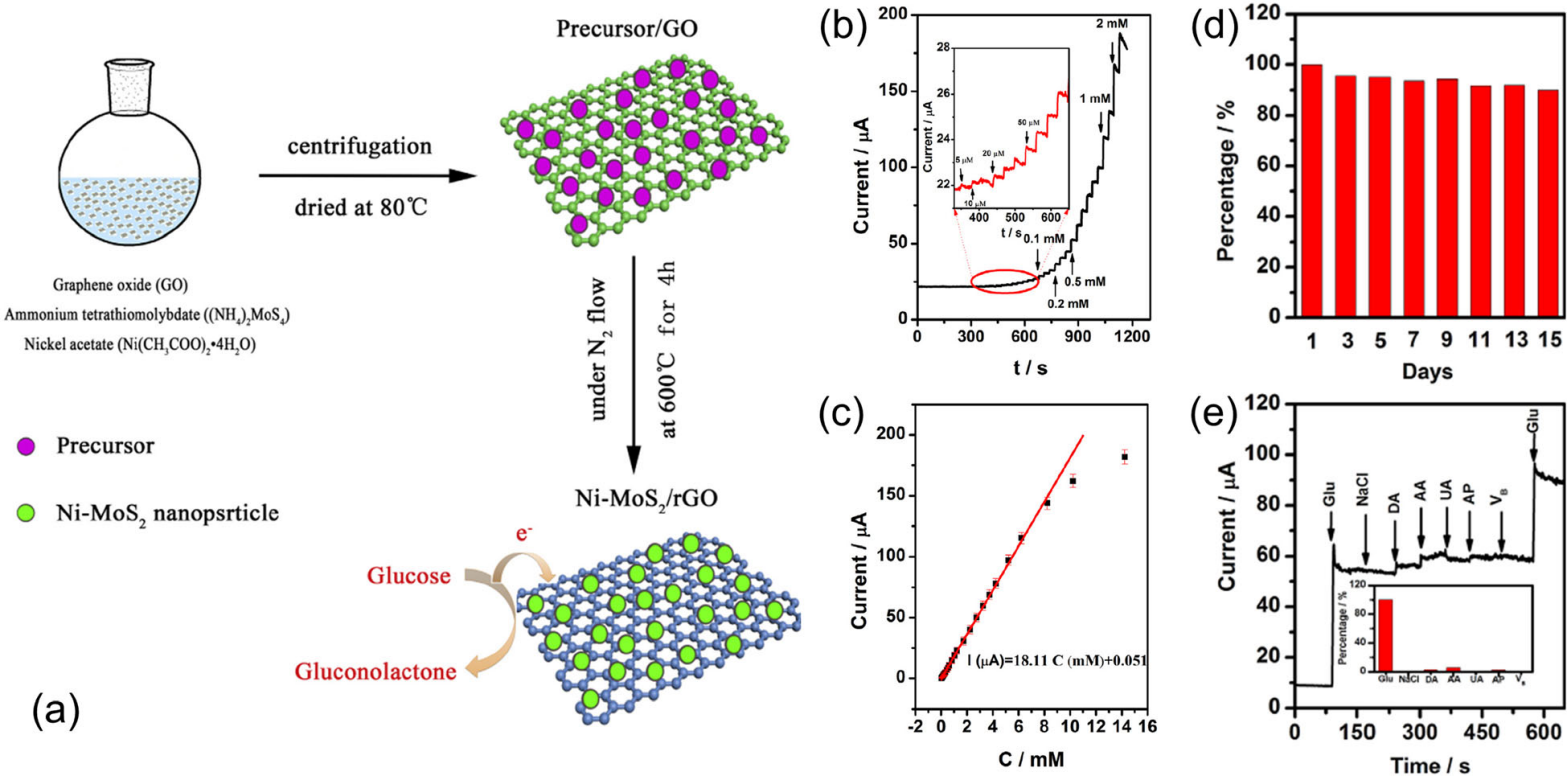
**a** Schematic of the synthesis of Ni-MoS_2_/rGO composites. **b** Amperometric response of a Ni-MoS_2_/rGO sensor to the successive addition of glucose. **c** The extracted response current to different glucose concentrations. **d** Stability test of the sensor by amperometric measurement for 15 days. **e** Comparison of the amperometric responses of 2.5 mM glucose and 0.1 mM interference chemicals. Reprinted from [25], Copyright 2017, with permission from Elsevier

The electrocatalytic activities of MoS_2_ can be further improved by hybridizing with graphene. The low intrinsic conductivity of MoS_2_ undermines its high catalytic activity. The charge transfers among MoS_2_ nanomaterials are slow in electrochemical reactions or general electronic applications. On the other hand, graphene has superior electrical conductivity and can serve as an immediate solution to slow electron transport in MoS_2_ nanomaterials [29]. Jeong et al. fabricated three-dimensional (3D) MoS_2_/graphene aerogel nanocomposites by a one-pot hydrothermal method [13]. Although enhanced electrocatalytic activities have been observed by using the 3D porous structure compared with the 2D reference sample, the use of glucose oxidase complicated the fabrication and faced the same issues of enzymatic sensors. Geng et al. synthesized Ni-doped MoS_2_ nanoparticles decorated on reduced graphene oxide (Ni-MoS_2_/rGO) by a facile and scalable method [30]. As shown in Fig. 1a, graphene oxide synthesized by the Hummers and Offeman method was mixed with CH_3_COOH and deionized water. Ni-Mo precursor solution was prepared by adding (NH_4_)_2_MoS_4_ and Ni(CH_3_COO)_2_·4H_2_O with different molar ratios into the graphene oxide solution. The Ni-MoS_2_/rGO suspension was obtained after centrifugation and drying at 80 °C. The collected Ni-MoS_2_/rGO suspension was then calcined for 4 h at 600 °C in N_2_ atmosphere. The obtained Ni-MoS_2_/rGO nanocomposites were used for non-enzymatic glucose sensing. Figure 1b shows the amperometric response of a sensor electrode modified by Ni-MoS_2_/rGO nanocomposites to the successive addition of glucose solution. A clear increase in current was observed after each addition of glucose. Additionally, the inset in Fig. 1b shows the sensor was capable of responding to a glucose concentration as low as 5 μM. The exacted current signal as a function of glucose concentration is plotted in Fig. 1c, which clearly shows a broad linear detection range of the sensor, 0.005–8.2 mM, well covering the typical human blood glucose level. The calculated sensitivity and detection limit is 256.6 μA mM^−1^ cm^−2^ and 2.7 μM (S/N = 3), respectively. Although the sensitivity and detection limit of the sensor is distinctly lower than the earlier ones, the linear detection range is well improved. The work further reported improved electron transport rates and electrocatalytic activity with a diffusion coefficient of 1.83 × 10^−3^ cm^2^s^−1^ and catalytic rate constants of 6.26 × 10^5^ cm^3^mol^−1^ s^−1^ by using the Ni-MoS_2_/rGO composites. As shown in Fig. 1d, when the sensor was stored under dry conditions, its current response to 1 mM glucose remains nearly unchanged for 15 days, showing good stability. The influence of common interference chemicals, NaCl, dopamine, uric acid, ascorbic acid, and V_B_, was also examined. The results are shown in Fig. 1e, and the impact of these chemicals on signal current is marginal. The current change caused by 0.1 mM of NaCl, dopamine, uric acid, ascorbic acid, and V_B_, a typical concentration of the interference chemicals in normal serum, is only 0.76%, 2.77%, 6.03%, 0%, 2.51%, and 0.63% of the current response to 2.5 mM glucose. Finally, the work demonstrated a good match between the measured concentration by the Ni-MoS_2_/rGO sensor and the reported hospital values, showing a great potential of the sensors for practical applications [30].

**Fig. 2:**
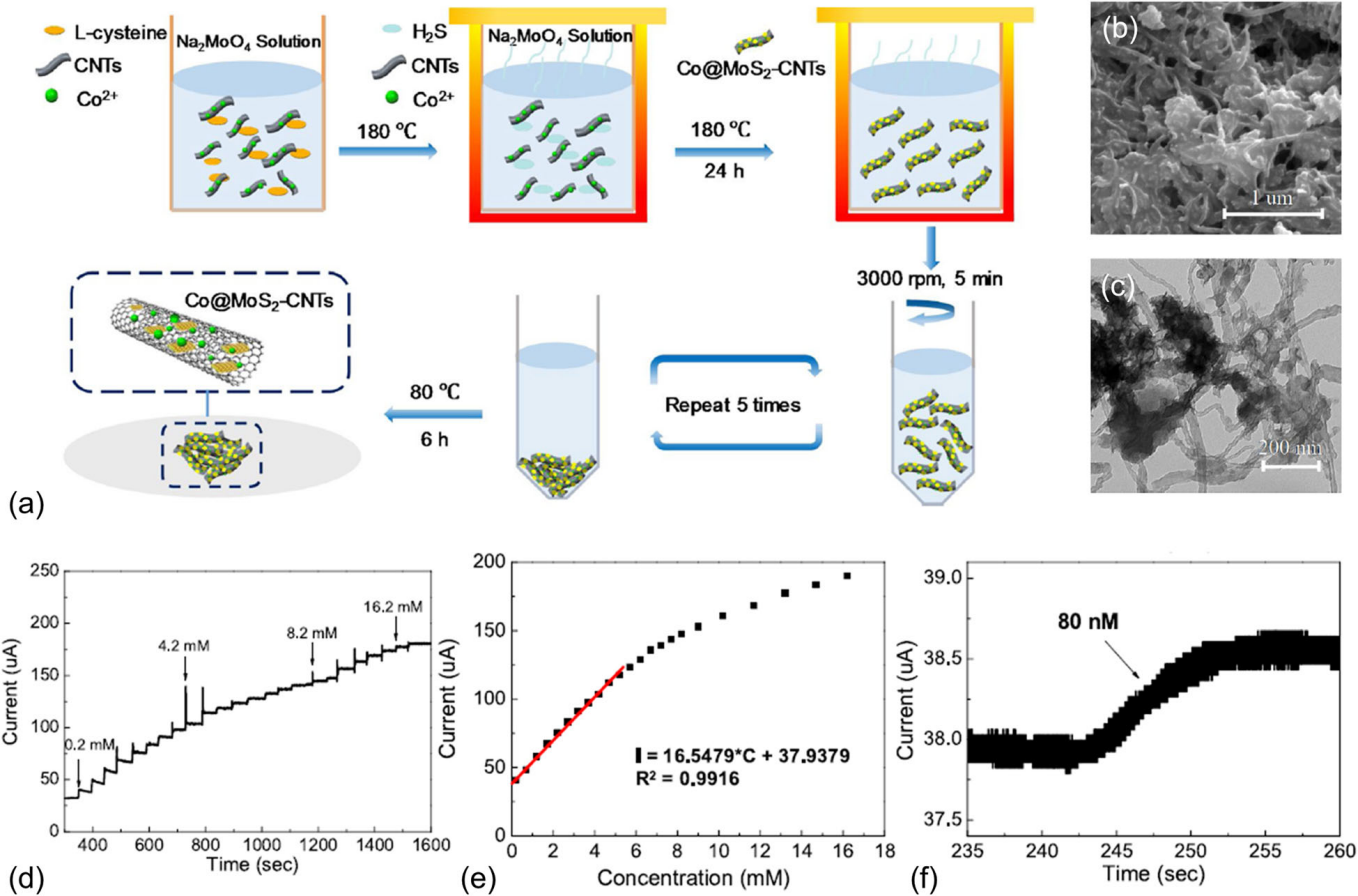
**a** Schematic diagram of the hydrothermal assembly of Co-MoS2/CNTs. **b** SEM and **c** TEM images of the synthesized Co-MoS2/CNTs. **d** Amperometric responses of the Co-MoS_2_/CNT sensor to the successive addition of glucose. **e** The extracted response current to different glucose concentrations. **f** Amperometric curve of the Co-MoS_2_/CNT sensor to 80 nM glucose. Reprinted from [29], Copyright 2019, with permission from Elsevier

An alternative way to improve the charge transfer in MoS_2_ nanocomposites is to hybridize with another highly conductive and bio-compatible carbon material, carbon nanotubes (CNTs). Meanwhile, this way can well limit the re-stacking of MoS_2_ nanomaterials, thereby providing more active reaction sites. CNTs have also been widely synthesized in 3D structures and applied in energy storage, energy harvesting, sensing, etc. [31–33]. Li et al. prepared a 3D nanocomposites of MoS_2_ nanosheets hybridized with cobalt oxide nanoparticles and CNTs [34]. The cobalt oxide nanoparticles were used to enhance electrocatalytic activities and the CNTs to improve conductivity. One-pot hydrothermal method used to synthesize the Co-MoS_2_/CNT nanocomposites is briefly shown in Fig. 2a. A mixture of CNTs, 0.1 mmol Co(CH_3_COO)_2_ 4H_2_O, 1.35 mmol Na_2_MoO_4_, and 7.5 mmol l-cysteine was transferred into a Teflon-lined stainless autoclave and kept at 180 °C for 24 h. The product was then cooled, centrifuged, and rinsed with deionized water and absolute ethanol. The cleaned Co-MoS_2_/CNT nanocomposites were finally dried in a vacuum oven at 60 °C for 6 h. The scanning electron microscopy (SEM) and transmission electron microscopy (TEM) images of the Co-MoS_2_/CNT nanocomposites are shown in Fig. 2b, c. Typical 3D bundled CNTs with diameters around 20 nm was observed. The TEM image clearly shows the hollow CNTs attached to MoS_2_ nanosheets. Such a structure serves as a highly conductive matrix to support MoS_2_ nanosheets and immobilize Co nanoparticles. Such the densely packed Co-MoS_2_/CNT nanocomposites not only provide a good amount of catalytic active edges but also allow efficient charge transfer during reactions. More importantly, the densely packed Co-MoS_2_/CNT structure and the fairly large interlayer spacing of 0.65 nm for MoS_2_ (larger than that of 0.34 nm for CNTs) effectively suppress re-stacking of MoS_2_/CNT composite. Typical cyclic voltammetry and amperometric measurements were carried out to examine the performance of the enzyme-free sensor made of Co-MoS_2_/CNT nanocomposites. The amperometric responses of Co-MoS_2_/CNT sensing electrode measured (at 0.65 V vs. Ag/AgCl) with successive addition of glucose are shown in Fig. 2d. A distinct step increase in response current to glucose addition was observed. A good linear detection range was achieved up to 5.2 mM, as shown in Figs. 2e. The calculated sensitivity is 131.69 μA mM^−1^ cm^−2^. Despite the relatively low sensitivity, an extremely low detection limit of 80 nM obtained (S/N = 3) from Fig. 2f.

**Fig. 3:**
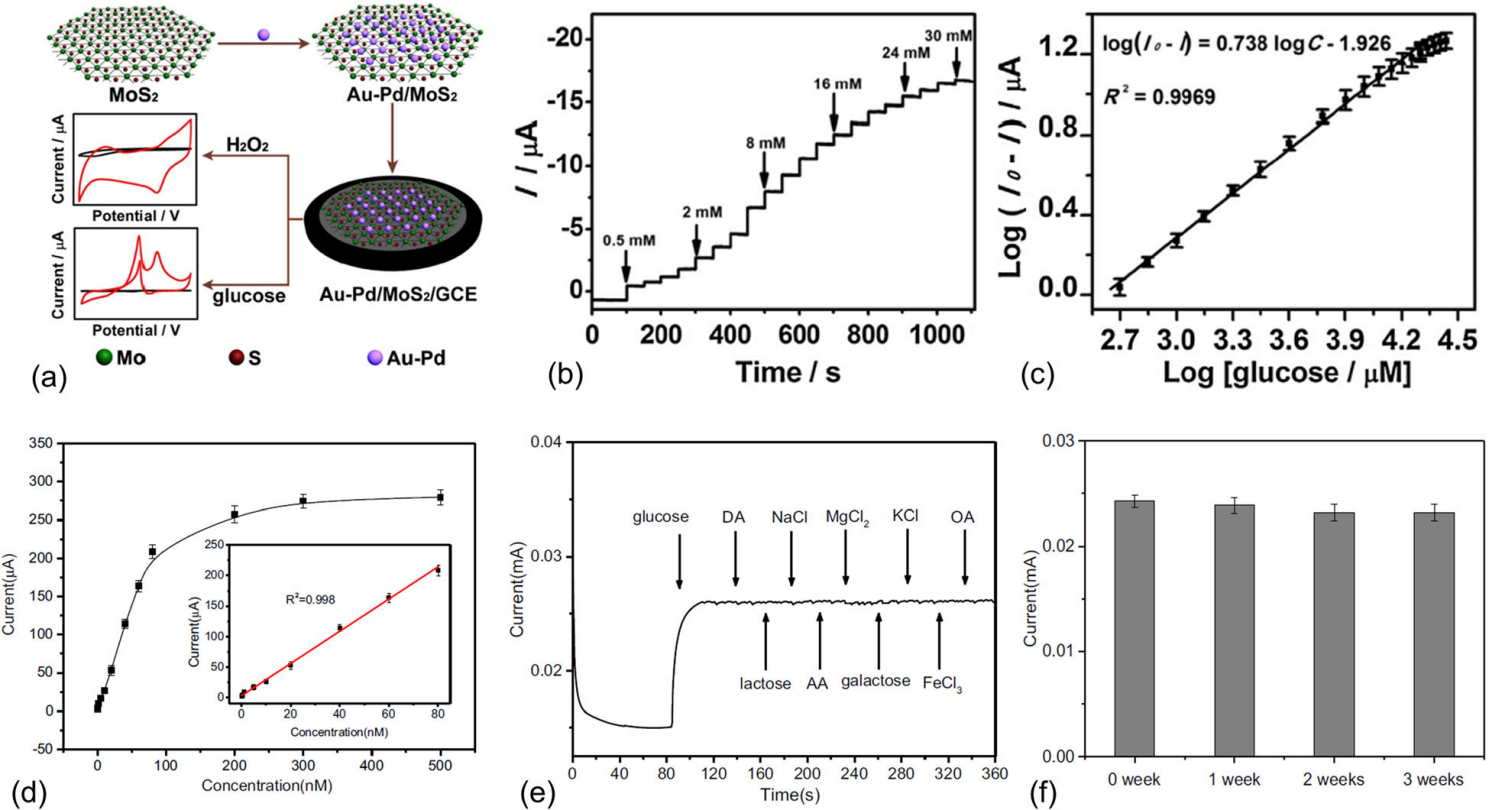
**a** Illustration of the synthesis of Au-Pd/MoS_2_ nanocomposites and assembly on a glassy carbon electrode for non-enzymatic electrochemical sensing of H_2_O_2_ and glucose. **b** Amperometric responses of the Au-Pd/MoS_2_ nanocomposite sensor to the successive addition of glucose. **c** The extracted response current to different glucose concentrations. Reprinted from [5], Copyright 2017, with permission from Elsevier

Similar to the synergistic effects displayed by metal-2D material hybrids, bimetallic alloys and nanostructures have also showed improved catalytic performance and showed good potential for many applications, including sensing [35], energy harvesting [36, 37], etc. Li et al. recently synthesized Au-Pd bimetallic nanoparticles for non-enzymatic hydrogen peroxide and glucose sensing [5]. The fabrication of the Au-Pd/MoS_2_ sensor electrode is illustrated in Fig. 3a. The MoS_2_ nanosheets were prepared by liquid exfoliation. Au-Pd bimetallic nanoparticles were synthesized by chemical reduction. The prepared Au-Pd/MoS_2_ nanocomposites were then deposited on a glassy carbon electrode for chemical sensing. As shown in Fig. 3b, good current steps were observed with a successive addition of glucose. The linear detection range measured as 0.5–20 mM is well beyond normal human blood glucose level (Fig. 3c). Instead of using conventional bimetallic nanoparticles which often made from expensive metals, Ma et al. designed a gold nanoparticle-polypyrrole (PPY) co-decorated MoS_2_ nanocomposite [38]. The metal/conductive polymer hybrids are also expected to improve the surface area and conductivity of a sensor electrode. Moreover, the use of conductive polymers can further reduce the cost of electrochemical sensors. The fabricated MoS_2_-PPY-Au/glassy carbon electrode showed an incredible low detection of 0.08 nM, nearly interference-free selectivity, and long stability over 3 weeks. However, the sensor sensitivity is only 37.35 μA·μM^–1^·cm^–2^ and the detection range is rather limited (0.1–80 nM).

In addition to metals, metal oxides of high catalytic activities have also been attempted for improved electrochemical catalysis. Apart from its high electrocatalytic activities, the low cost of metal oxides is another advantage that cannot be overstressed for low-cost electrochemical sensors. Among various metal oxides, Cu_2_O nanomaterials with different morphologies are promising for catalysis in various applications. Fang et al. have been studied MoS_2_ decorated with Cu_2_O nanoparticles for non-enzyme glucose sensing [39]. The amperometric measurements of the Cu_2_O/MoS_2_ hybrid-modified electrode show a good linear range from 0.01 to 4 mM. The extracted detection limit is about 1 μM. The sensitivity was calculated as high as 3108.87 μA mM^−1^ cm^−2^, which is higher than most MoS_2_-based non-enzyme glucose sensors. The results also indicate a good potential of metal oxides for low-cost non-enzyme glucose sensors. The comparison of the enzyme-free glucose biosensors based on MoS_2_ nanocomposites is presented in Table 1.

**Table 1:** Comparison of the enzyme-free glucose biosensors based on MoS_2_ nanocomposites

Sensing materials	Applied potential (V vs. Ag/AgCl)	Linear range (mM)	Detection limit	Sensitivity (μA mM^−1^ cm^−2^)	Response time (s)	Reference
Cu-MoS_2_ hybrid	0.65	0–4	–	1055	–	[26]
Ni-MoS_2_ hybrid	–	0–4	0.31 μM	1824	< 2	[27]
AuNPs/MoS_2_	0.93	0.00005–0.1	5 nM	–	2	[24]
MoS_2_ microflowers	0.35	0–30	–	570.71	–	[25]
MoS_2_-Au/Pt	0.6	0.01–19.07	0.39 mM	142.68	–	[35]
Au-Pd/MoS_2_	0.3	0.5–20	0.40 mM	184.9	–	[5]
Ni-MoS_2_ /rGO	0.55	0.005–8.2	2.7 μM	256.6	< 2	[30]
AgNPs/MoS_2_	0.31	0.0001–1	0.03 μM	9044.6	–	[28]
Cu_2_O/MoS_2_	0.4	0.01–4.0	1.0 μM	3108.87	< 2	[39]
Bilayer MoS_2_	–	30–300	300 nM	260.75	< 1	[40]
MoS_2_-PPY-AuNPs	0.45	0.1–80 nM	0.08 nM	37.35	< 2	[38]
Co@MoS_2_/CNTs	0.65	–	80 nM	131.69	4.5	[34]

## High-Sensitivity Glucose Detection Using MoS_2_ Field-Effect Transistors

**Fig. 4:**
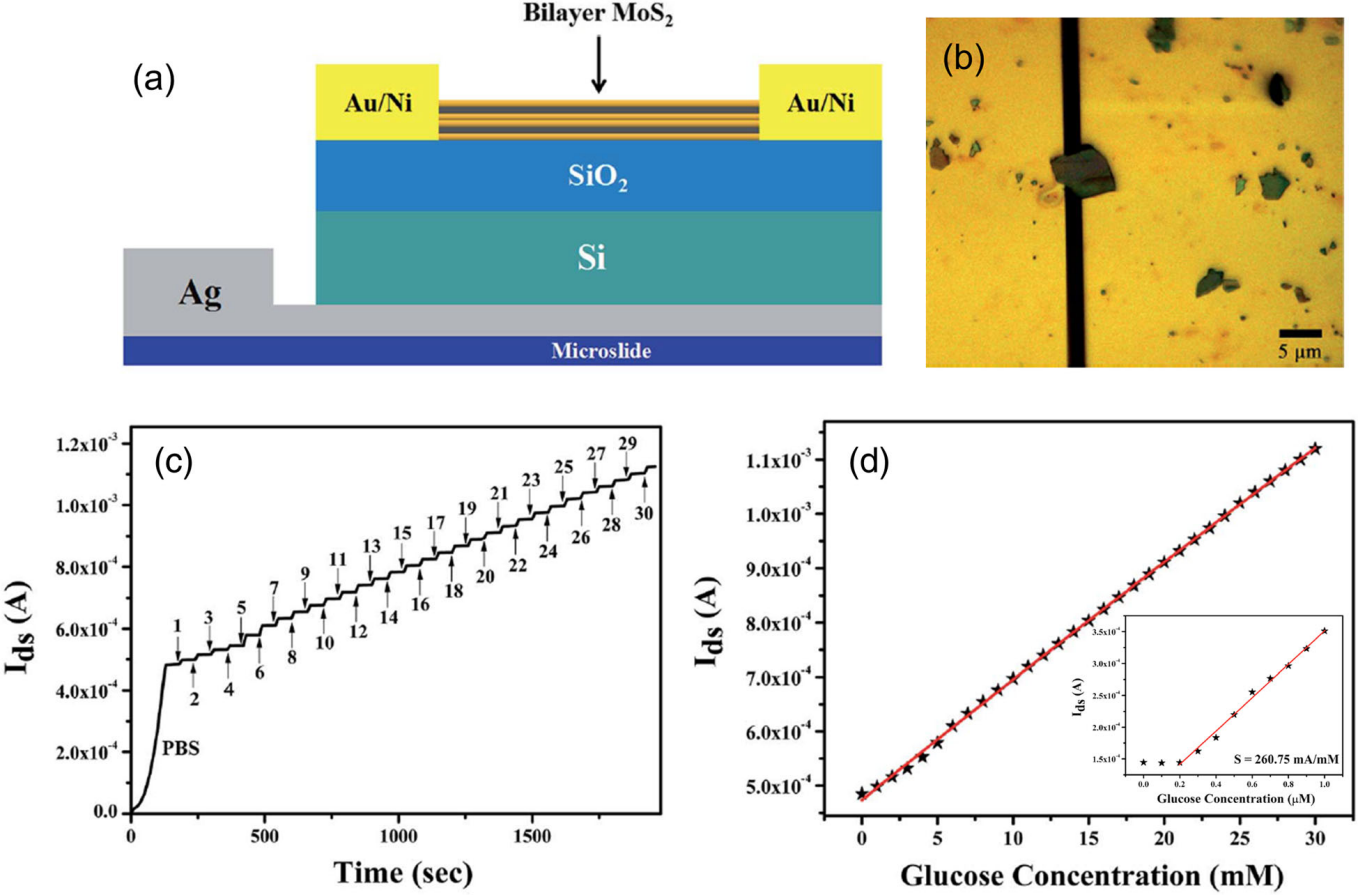
**a** Schematic of the back-gated MoS_2_ transistor. **b** Optical microscopy image of the MoS_2_ channel material between the source and drain electrodes. **c** The real-time current responses to different glucose concentrations. **d** The response current *I*_ds_ of the MoS_2_ FET as a function of glucose concentrations, from 0 to 30 mM. The inset shows the response current extracted for lower glucose concentrations, from 0 to 1.0 μM. Reprinted under CC BY-NC 3.0 from [44]

MoS_2_ field-effect transistors (FETs) possess a number of advantages, such as high switching current ratio, low leakage current, small subthreshold swing, and high mobility [41, 42]. Thanks to its excellent electronic properties and mechanical robustness, MoS_2_ transistors show good promise for low energy, low cost, and wearable electronics [43, 44]. Biosensors, photodetectors, gas sensors, and their flexible counterparts based on MoS_2_ transistors have recently been reported [45]. The advantages of MoS_2_ transistors make these sensors highly sensitive, low in power consumption, portable, etc. MoS_2_ FETs have been reported as various sensors for humidity, H_2_O_2_, NO, NO_2_, NH_3_, DNA, etc. [46–49]. Shan et al. reported the first MoS_2_-based field-effect transistor for glucose detection [40]. As shown in Fig. 4a, a back-gate MoS_2_ FET was fabricated on a SiO_2_/Si substrate. Source and drain electrodes were patterned by photolithography and e-beam lithography. Au/Ni (70 nm/10 nm) contacts were deposited by evaporation. It should be noted that the MoS_2_ channel material of about 2 μm × 3 μm was mechanically exfoliated and transferred to the pre-patterned electrodes, as displayed in Fig. 4b. The fabricated transistor was placed in a sample cell and tested.

The measured *I*_ds_–*V*_ds_ curves with different concentrations of glucose solutions clearly showed increases of source-drain current with increase of glucose concentration. It should be noted that GO_*x*_ enzyme was added to the glucose concentration. Therefore, the sensing is not completely enzyme-free. The enhanced channel current upon increasing glucose concentration was attributed to the enzymatic glucose oxidation. The electrons produced from the reaction were transferred to the n-type MoS_2_ channel and hence increased its conductivity. To illustrate the sensor response to glucose, the real-time *I*_ds_ measurement was carried out with successive addition of different concentrations of glucose, as shown in Fig. 4c. The measurements started with a pure PBS solution and a higher concentration solution with 1 mM more glucose placed the previous one every minute. The source-drain current response to different concentrations of glucose is plotted in Fig. 4d. Clearly, the MoS_2_ FET sensor shows a very large linear range for glucose detection, up to 30 mM. The tests were repeated for a low concentration of glucose solutions to probe the detection limit and sensitivity of the MoS_2_ FET sensor. As shown in the inset of Fig. 4d, the sensor can clearly detect the presence of glucose with a concentration as low as 300 nM. The sensitivity of the MoS_2_ FET glucose biosensor is calculated to be 260.75 mA mM^−1^. In addition to high sensitivity and low detection limits, the device also showed high stability for up to 45 days. However, the current device has to involve the addition of GO_*x*_ enzyme in the glucose solution under test, which makes it less practical for portable applications.

## Conclusion and Perspectives

This mini-review presents the recent efforts made towards developing enzyme-free biosensor-based MoS_2_ nanocomposites. These publications all presented facile and low-cost means to high-performance glucose sensors, in terms of sensitivity, linear detection range, and detection limit. These studies undoubtedly open new opportunities towards low-cost and sensitive glucose sensors. The advancements are largely depending on the recent progress made in the synthesis of novel nanocomposites of 2D materials, metallic nanomaterials, and catalytic oxide nanoparticles. It can be expected that more efforts would be invested in this direction, and the experience accumulated is highly beneficial to future studies on related materials for sensing applications.

However, at the same time, one should realize that many efforts are yet required for clinical or any other practical applications. The stability and reproducibility of these devices are yet to be improved. Either limited storage time or in dry conditions were so far used. Secondly, the chemical synthesis methods are facile and low cost, but whether the methods are scalable remains unclear. New techniques, such as inkjet printing, may be used for repeatable large-scale fabrication of sensors. Although MoS_2_-based electrochemical sensors show competitive performance compared with the carbon material-based counterparts, the advantages, e.g., catalytic edge sites of MoS_2_, are not substantial. There is clearly a lot of room to really take advantage of the unique properties of MoS_2_ for further improvements in non-enzymatic glucose sensing. Furthermore, the development of flexible glucose biosensor-based MoS_2_ nanocomposites is important for flexible sensing in healthcare and should be more competitive in the market, which will surely become a research hotspot in the future.

Finally, MoS_2_ FET-based sensors show excellent performance in glucose sensing. Given the recent development of MoS_2_ FETs, this direction seems very promising in developing low-cost glucose sensors and other types of chemical sensors. It should be restressed that the current work reported on MoS_2_ FET glucose sensors was only functional to GO_*x*_-doped glucose solution. Future work needs to find alternatives to avoid the use of GO_*x*_ for more practical deployment of MoS_2_ FET glucose sensors.

## Data Availability

Not applicable.

## Abbreviations

2D: Two-dimensional
3D: Three-dimensional
CNT: Carbon nanotubes
CTAB: Cetyltrimethylammonium bromide
FETs: Field effect transistors
GO*x*: Glucose oxidase
Initials: Full name
MoS_2_: Molybdenum disulfide
Ni: Nickel
SEM: Scanning electron microscopy
TEM: Transmission electron microscopy
TMDs: Transition metal dichalcogenides
